# Some Welfare Assessment Traits and Quantitative-Qualitative Milk Parameters as Affected by Supplementary Feeding at Milking and Parity in Anatolian Buffalo Cows

**DOI:** 10.3390/ani14060956

**Published:** 2024-03-19

**Authors:** Ahmet Akdağ, İbrahim Cihangir Okuyucu, Hüseyin Erdem, Ertuğrul Kul, Nuh Ocak

**Affiliations:** 1 Department of Animal Science, Faculty of Agriculture, Eskisehir Osmangazi University, 26480 Eskisehir, Turkey; ahmeta@ogu.edu.tr; 2 Department of Animal Science, Faculty of Agriculture, Ondokuz Mayis University, 55139 Samsun, Turkey; cihangir.okuyucu@omu.edu.tr (İ.C.O.); nuhocak@omu.edu.tr (N.O.); 3 Department of Animal Science, Faculty of Agriculture, Kırşehir Ahi Evran University, 40100 Kırşehir, Turkey; ertugrul.kul@ahievran.edu.tr

**Keywords:** multiparous buffalo cow, temperament, udder hygiene, body condition, milk production, milk quality, feeding

## Abstract

**Simple Summary:**

A critical issue is the significant decrease in productivity and heightened consumer awareness caused by the inadequate welfare of confined lactating water buffaloes (*Bubalus bubalis*). Providing supplementary feeding during milking (SFAM) helps alleviate the adverse effects of stall confinement on milk quality by enhancing welfare indicators like udder hygiene, body condition and milking behaviour. Additionally, SFAM has comparable benefits for second and third-calving buffalo cows. Implementing SFAM for barn-confined buffalo cows improves production outcomes, regardless of parity, positively impacting consumer perception.

**Abstract:**

This study aimed to evaluate whether supplemental feeding at milking (SFAM) positively influences the quantitative−qualitative milk parameters due to improving some welfare assessment traits of multiparous Anatolian buffalo cows confined in semi-open free-stall barns. A total of 76 Anatolian buffalo cows at approximately 90 days in milk were selected to encompass four groups (OSF-2nd, NSF-2nd, OSF-≥3rd and NSF-≥3rd), considering offering (OSF) or not (NSF) supplemental feed at milking and the parity (2nd) and (≥3rd). Data of evaluated variables such as the following ones—(i) subjectively scored welfare assessment traits (temperament, udder hygiene and body condition), (ii) milk yield per milking (MYM), (iii) milk components, and (iv) milk physical traits—were analysed using a linear mixed model and principal component (PC) analysis. The OSF improved the temperament, udder hygiene and body condition scores compared to the NSF. The MYM, the fat content and the fat-to-protein ratio of the OSF were higher than those of the NSF, but milk mineral and electrical conductivity of the OSF were lower than those of the NSF. The parity of cows did not affect the evaluated variables. Four parameters (milk density value and lactose, solids-not-fat and protein contents) could be identified in the PC2 versus PC1 plot. In conclusion, the SFAM enhanced the milk yield and qualitative milk parameters due to improving the welfare status of indoor buffalo cows, regardless of parity.

## 1. Introduction

Water buffaloes (*Bubalus bubalis*) are commonly raised under extensive pasture-based farming conditions. However, increasing interest in buffalo dairy products has accelerated the transition from traditional to modern production systems in many countries, including Turkey [1]. The transition to modern or intensive production systems has changed consumers’ perceptions of food quality and increased their interest in buffalo milk. Consumers often associate food quality with the nature of the products, their safety and the welfare of the animals from which they are produced [2].

The same modern systems used for dairy cows do not meet the physiological and well-being requirements of buffaloes that move away from natural habitats [2,3,4,5] due to critical stressors (human interactions, management routines such as handling, feeding, milking and mechanisation). Modern management practices and physiological load from mechanic milking procedures have negatively impacted the productivity and welfare of buffaloes, which are sensitive to the environment and have quick behavioural responses [4]. Therefore, extensive attention has recently been paid to the buffalo response to these changes [2], the relationships between milking temperament and personality, and some productive indicators such as milk yield per milking (MYM) [4,5]. This situation dramatically reduces the welfare of buffaloes [4,6,7] and subsequent milk yield and quality of milk and its products [2,5,8]. Moreover, the technological characteristics of milk and its products, which are of great importance for the buffalo dairy industry, are directly affected by the milk quality, such as chemical and physical traits [9,10].

In intensive buffalo production systems, social isolation causes a change in the environment-, animal- and both-based welfare criteria (udder hygiene, body condition and temperament), because buffalo cows are social animals [11]. Moreover, because production and personality are negatively related overall, docile individuals have been expected to be more productive in social isolation [12]. Accordingly, the welfare criterion is a principal concept that must be considered in developing buffalo production systems despite the on-farm animal welfare assessment being time-consuming and costly [4,7]. On-farm animal welfare assessments have been evaluated against compliance and relationships with resource-based and animal-based welfare criteria and productivity [4,13]. Some studies [4,5,8,14] have evaluated the relationships between welfare assessment traits such as udder hygiene score (UHS), temperament score (TS), body condition score (BCS) and quantitative−qualitative milk parameters, including freezing point (FP) and electrical conductivity (EC). These studies showed that the MYM and some milk characteristics have enhanced as the welfare indicators related to barn conditions and the strategies of milking and feeding for lactating buffalo cows have improved. On the other hand, the contamination level in the cows’ bodies may increase due to restricting the cows’ movement area and excessive wetness or manure accumulation in overcrowded paddocks [15]. In addition, cows lying and resting on unsuitable bedding and ground and their rapid movement for milking may expose them to manure and mud [4]. Such management and barn conditions cause high udder contamination or UHS, negatively affecting milk yield and qualitative milk parameters, including milk EC and FP values [5,15,16,17].

Since buffaloes are challenging to milk under confinement, milking management is one of the most critical activities in the milk production chain [18]. Furthermore, maintaining a balance between milking welfare and productivity is essential due to increased productivity while reducing feed and labour costs [5,19]. The restlessness of dairy animals during mechanical milking is associated with unsuitable milking procedures and attitudes and behaviour of stockpersons towards cows [2,3]. Compared with resource-based welfare indicators, animal-based indicators are more closely linked to animal welfare, because they measure animals’ actual state [14], regardless of housing and managed conditions. Supplementary feeding at milking (SFAM), which is frequently involved in dairy cattle due to it encouraging calmness and allowing them to get used to milking procedures [20,21,22], can also be applied and beneficial to buffalo cows [4,23,24]. Indeed, better health, MYM, milk quality and economy have been obtained in indoor primiparous buffalo cows that are offered roughage with concentrates such as total mixed ration (TMR) or partial mixed ration at milking [5].

Water buffaloes, including Anatolian buffaloes, are very sensitive to environmental stimuli before and during milking [25], including changes in milking routines [4,5,26]. Moreover, buffalo cows in the 2nd parity exhibit more nervous behaviour during milking [4] than those in ≥3rd parity. Therefore, SFAM impacts can differ among buffalo cows in the 2nd and ≥3rd parity. Recently, several studies [4,5,25] on milking management in buffalo cows have focused on the association between welfare criteria such as TS, UHS and BCS with milk yield, as well as milk’s chemical composition (fat, protein, solids-not-fat (SNF), lactose, minerals and fat-to-protein ratio (FPR)) and physical properties (milk density, FP and EC values), which are crucial for the dairy buffalo industry [27,28]. However, limited information exists on the relationships between milking management and welfare criteria in multiparous buffalo cows with different parity [5]. Therefore, this study aimed to evaluate whether SFAM positively influences the quantitative−qualitative milk parameters such as MYM, the milk composition and physical traits due to improving some welfare assessment traits such as TS, UHS and BCS of Anatolian buffalo cows in the 2nd and ≥3rd parity confined in semi-open free-stall barns. The other aim was to underline the relationships between the milk quality parameters affected by SFAM and parity using a chemometric approach.

## 2. Materials and Methods

### 2.1. Feeding, Housing and Management of Cows

All the experimental procedures and welfare protocols were performed according to the guidelines recommended for applied animal behaviour research on protecting animals used for experimental and other scientific purposes [29]. This observational study was conducted with multiparous Anatolian buffalo cows (body weight of 480 ± 33 kg) at a commercial farm having an approximate herd size of 140 heads in Samsun, Turkey. Cows used in the study were selected from cows kept in a semi-open free-stall barn with a concrete floor, calved within two months (October and November) and in the 2nd and ≥3rd (3rd, 4th, and 5th) parity. These cows were selected to cover supplementary feeding, which was offered (OSF) or not (NSF) at milking, with 19 cows each. Thus, 76 healthy-milking Anatolian buffalo cows at approximately 90 days in milk were allocated to encompass four groups (OSF-2nd, NSF-2nd, OSF-≥3rd and NSF-≥3rd), considering SFAM (OSF or NSF) and the parity (2nd) and (≥3rd).

Before and after milking, all cows were not grazed in the pasture and were fed ad libitum with a TMR (Table 1) in the barn. Additionally, cows had free access to fresh water throughout the day. At milking, cows in the OSF group were offered a TMR of 2 kg without considering feed requirements for energy and crude protein. Each cow consumed the offered feed, until milking was completed.

### 2.2. Scoring, Measurements and Milk Analyses

All scoring and measurements in the study were performed without interfering with routine herd management practices on the farm. The welfare assessment traits (TS, UHS and BCS) were assessed twice for two consecutive days at 7-day intervals. A trained classifier scored these subjective assessment-based well-being traits. Temperament and body conditions were scored during the morning milking. The UHS was performed immediately after milking. The welfare assessment traits were assessed separately for each buffalo cow, as explained previously [4], based on the scoring systems in Table 2.

Cows were milked once daily in the morning (between 05:00 a.m. and 08:00 a.m.) using a portable milking machine, which worked at a 44 kPa vacuum (PLS-2/1, Sezer, Bursa, Turkey). Two cows were milked simultaneously by the same stockperson using two machines. Routine milking practices on the farm were not changed. Calves were allowed to suckle naturally on their dam for approximately 1 min to stimulate the milk let-down. At each milking, the cows’ teats were washed by the stockperson with warm water and dried with hygienic milking materials (cloth). After the milking procedure, no iodine teat dip was performed. Milking was performed after the machine’s milking cups (clusters), fitting for the teat anatomy, and was attached to the cows’ teats. Until the milking processes were completed, the calves were kept next to their dams, where they could not reach the udder during milking. After milking, the calves were kept in their next dams (about one hour) to suck the remaining milk [13].

To determine the MYM (kg per cow), the milk collected separately for each cow was transferred to an empty tared bucket and weighed using a digital scale. To assess milk quality traits, milk samples of approximately 50 mL were collected in plastic milk tubes belonging to each cow and sent to a milk testing laboratory (Department of Animal Science, Faculty of Agriculture, Ondokuz Mayıs University) in a cold chain bag (+4 °C) on the same day for milk components (fat, protein, lactose and mineral) and physical traits (density, FP and EC) analysis. However, the milk samples were gently mixed and heated in a warm water bath to 32 °C for 15 min before analysis. The milk fat, protein, lactose, SNF and mineral contents (%), density (mg/mL) and FP (°C) of the milk samples were determined using a LactoStar milk analyser (calibrated with appropriate buffalo standard, Funke-Gerber, Labortechnik GmbH, Ringstraße, Berlin, Germany) equipped with a conductometric sensor. Fat and protein contents were used to calculate the FPR. The milk EC (mS/cm) was determined using a portable EC meter (Mettler Toledo, GmbH, Heuwinkelstrasse, Nänikon, Switzerland). At least three replicates were performed in these analyses.

### 2.3. Statistical Analysis

The data were analysed using the SPSS software program (version 21.0, SPSS Inc., 183 Chicago, IL, USA). Before analysis, the assumption of the normality test (Kolmogorov−Smirnov test) and the homogeneity test (Levene’s test) were performed on the MYM, fat, SNF, protein, lactose, mineral, density, FP, EC and FPR values (*p* > 0.05). The experimental unit was the cow (n = 19 per treatment) for all data. A linear mixed model was built with SFAM, parity, an interaction term (SFAM × Parity) as fixed effects, farm as random intercepts and robust standard errors. Based on Duncan’s multiple comparison test, the mean differences were accepted as significant when *p* < 0.05. In addition, the UHS, BCS and TS data were analysed using the Kruskal−Wallis test, a non-parametric test. Principal component analysis (PCA), a chemometric approach, was performed to understand the relationship between milk quality traits. The results were presented visually by reducing dimensionality using two-dimensional scatter plots. We interpreted it as a positive correlation when two variables operated in the same quadrants. In contrast, when two variables move in opposite quadrants, we interpreted it as a negative correlation.

## 3. Results

The welfare assessment traits (TS, UHS and BCS) were affected by SFAM (*p* < 0.001), whereas they were not affected by parity and the interaction between SFAM and Parity (Table 3). The TSs and the UHSs of the NSF cows were higher than those of the OSF cows. However, the BCSs of the NSF cows were lower than those of the OSF cows (*p* < 0.001).

The MYM, the fat percentage and the FPR that were affected by the SFAM (*p* < 0.001) were higher in the milk from the OSF group than those from the NSF group (Table 4). Compared with the OSF cows, the NSF cows produced milk with higher mineral percentages and EC values (*p* < 0.001). The interaction between the parity and SFAM × Parity did not affect the MYM, milk components and physical traits.

**Figure 1:**
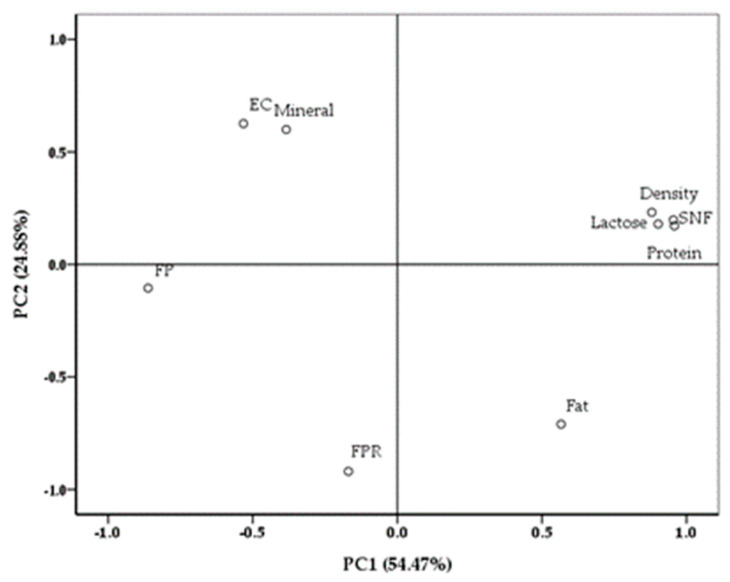
Loading principal components (PC1 and PC2) plots for milk chemical and physical traits. FP—freezing point; FPR—fat-to-protein ratio; SNF—solids-not-fat; EC—electrical conductivity.

The most important principal components (PC) generated from milk chemical and physical traits and their statistical loadings are presented in Figure 1. The PC1 and the PC2, which accounted for 79.35% of the total variation in the data set, had the highest eigenvalues of 4.90 and 2.23, representing 54.47% and 24.88% of the total variance, respectively.

Even though milk quality traits were distributed to all quadrants of PCA, the scores corresponding to the PC1 and the PC2 show that it had three groups. Group 1 was composed of traits with positive loadings for the PC1 and the PC2 (SNF [0.950 and 0.223], protein [0.953 and 0.196], lactose [0.896 and 0.204] and density [0.861 and 0.180]). Group 2 included milk fat with a positive score for the PC1 and a negative score for the PC2 [0.587 and −0.693]. Finally, group 3 was composed of milk traits with negative scores for the PC1 (FP [−0.857 and −0.123], mineral content [−0.387 and 0.598], EC value [−0.546 and 0.611] and FPR [−0.142 and −0.922]). Four parameters (milk density, lactose, SNF and protein percentages) could be identified in the PC2 versus PC1 plot.

Different from in PC1, in PC2, no mutual relationship was observed between fat content and other nutritional traits. In contrast, milk mineral content and EC value were related to opposite quadrants, indicating that these were negatively correlated. This relationship may represent a decrease in milk fat content when milk minerals and EC value increase. There were also similar relationships among the parameters (milk FP and FPR) in group 3 and those (milk lactose, protein, SNF contents and density value) in group 1, since these two groups of parameters were located in opposite quadrants.

## 4. Discussion

In the present study, regardless of parity, the OSF calmed down the multiparous Anatolian buffalo cows, restless during machine milking [4], and improved their UHS and BCS. Our outcomes supported the notifications that buffalo cows classified as docile had higher milk yields than those classified as nervous [4,5,13]. Indeed, we observed that high TS reflects the aggressive behaviour of buffalo cows during milking, which resulted in an increased contamination level of cows or a high UHS [4,5]. Moreover, the correlation between the UHS and the BCS with the TS in the primiparous Anatolian buffalo cows is significant [5]. As reported previously [23,35,36], the SFAM significantly improves milking performance, indicating that it is beneficial to improve the milking temperament, as well as the UHS and the BCS of buffalo cows. A previous study has confirmed that the milking temperament and subsequently udder hygiene and body condition of Anatolian buffalo cows worsen as the frequency of stress or fear indicator behaviours such as kicking, stepping, urinating, defecating and pulling the teat cup off during milking increase [4]. In addition, the hygiene of cows and the barn is related to feeding management, which is an integral part of herd management [4,35]. This result supports the idea that the SFAM reduces their interest in the environment by distracting cows until milking is completed [5]. As such, the SFAM may be beneficial in improving environmental factors that adversely affect buffalo cows’ milking temperament and udder hygiene, as in previous studies [5,23,24].

Because the mature cow weight is affected by the BCS, which is associated with muscle mass and fat accumulation, the BCS is a critical parameter to consider in breeding goals as an indicator of cow efficiency [37]. The BCSs (2.61 to 2.98) of multiparous cows were found to be lower than the ideal values (3 to 3.25) due to the cows’ probably being in the early lactation period [33,34]. Furthermore, early lactation cows (approximately 90 days) can be unsuitable for monitoring body conditions and detecting differences in welfare due to the intense mobilisation of body fat [30,38,39]. This case may be related to calmer cows having a higher BCS than nervous animals [40], because the feed efficiency of calm cows is associated with increased body fat reserves [40,41]. Although the amount of supplemental feed offered at the milking was insufficient to meet all dietary requirements, it may have contributed to meeting the cows’ nutritional requirements [5,21]. Thus, the SFAM may have preserved body fat reserves indicative of the BCS by improving net energy balance in early lactation [5,21]. These findings indicate that NSF cows were more nervous and, as a result, had lower MYM and milk quality than OSF cows because increased metabolic stress during machine milking negatively affects energy metabolism and metabolic stress indicators, such as cortisol and malondialdehyde [42,43]. This case indicates that buffalo cows can have individual personality traits that affect their behaviours and thus regulate how they meet these needs in addition to their metabolic and physiological needs regarding precision feeding, as in dairy cattle [21,43]. Therefore, regardless of parity, the OSF probably positively affected the mechanism of regulating indoor buffalo cows’ temperament, body conditions and nutritional requirements, which are sensitive and aggressive to machine milking [5]. In addition, our results indicate that the SFAM can be an effective milking management practice in meeting the expectations of farmers and consumers in terms of increasing the well-being of buffalo cows and, subsequently, improving quantitative−qualitative milk parameters, food safety [18,44,45,46] and economy [4,5,44].

The fact that the parity did not affect the TS, the UHS and the BCS of the multiparous buffalo cows indicates that these cows have the advantage of being accustomed to milking processes from previous lactation periods. Similar results regarding animal welfare scores were found in studies on Anatolian buffalo cows [47] and Simmental cows [48]. On the contrary, the milking temperament of Anatolian buffalo cows with ≤2 parity has been higher than those with ≥4 parity at 6 and 30 days in milk [4]. In addition, Antanaitis et al. [30] noted that older Holstein cows have a calmer milking temperament compared to younger cows. The impact of parity on quantitative−qualitative milk parameters was quite variable, as observed in the literature; Verma et al. [49] did not determine significant changes in these parameters, as in our study, while others observed an increase in milk components and milk yield [50,51]. The efficiency of the milking process in buffaloes is more affected by anatomical factors, especially teat morphology [26], and physiological differences are found between these species than between dairy cattle [43,52,53].

Milk quality, directly impacting the technological characteristics and pricing of milk, is crucial for the buffalo dairy industry [10], because the quality of milk products is affected directly by milk’s chemical composition and physical traits [9]. Animal welfare, which influences milk quality, should be enhanced to improve buffalo milk products’ quality and shelf life, as in previous studies [2,4,6,8]. In the present study, SFAM improved the studied animal assessment traits and positively affected MYM and some milk quality characteristics, such as fat and FPR. However, it reduced mineral percentage and EC value due to their positive correlation with UHS [4,5,17,23]. Our results regarding the quantitative−qualitative milk parameters, except for milk FP, were very close to the normal range reported for Anatolian buffalo [5,54] and Italian Mediterranean buffalo cows [55]. The FP values reported here are lower than those stated for Anatolian buffaloes (−0.65 to −0.48 °C) [54] and Italian Mediterranean buffaloes (−0.574 to −0.512 °C) [55]. This situation may be related to the following situations: (i) the fact that it was determined in fresh milk that was milked mechanically in the morning; (ii) the negative correlation between the FP value and the milk density values and protein, SNF, mineral and lactose contents; and iii) the differences that resulted from calibration procedures in the automated analytical techniques (e.g., LactoStar device versus MilkoScan) [5,54,55]. Indeed, Ceniti et al. [55] noted that in the milk samples collected through manual milking in the morning and evening, the milk FP value showed a negative correlation with milk protein and fat contents while presenting a positive correlation with lactose. Our study determined a negative correlation between the milk fat percentage and the EC value, as reported by Vilas Boas et al. [56].

Confirmed positive associations of lactose content in milk with reproductive success and udder health are essential for the profitability and sustainability of dairy buffalo farms [57,58]. FPR, lactose percentage and EC value are among the indicators of several welfare evaluation criteria for cows, such as temperament, udder health, fertility and metabolic status [30,59]. Additionally, it has been emphasised that changes in milk density are closely related to the SNF and fat percentages of the milk [28]. Based on the PCA result, the confirmed relationships among quantitative−qualitative milk parameters may explain the mechanisms of action and reflections of welfare assessment traits such as milking temperament, udder hygiene and body condition [28,30,59]. The discrepancy between the results of current and previous studies may be explained by differences in milking practices (pre-stimulation, supplementary feeding and oxytocin injection at milking), parties, lactation stages, milking time, bread and body weight of dairy animals and feed or TMR [26,43,54,55].

Pre-stimulation is less critical for milk ejection in restless dairy cattle [43], whereas pre-stimulation alone is generally insufficient for this aim in dairy buffaloes with nervous temperament [26]. However, feeding during milking positively influences milking characteristics (milking time, milk flow rate and MYM) and behaviour indicators (milking temperament, udder hygiene, social interactions, total standing and ruminating) in cattle [60,61] and buffalo cows [4,5], as in the current study. Machine milking, a source of stress for dairy cattle [21] and buffaloes [4,5], completed without affecting the cows’ physical, physiological and emotional state, indicates a decrease in the level of stress hormones in the blood and, thus, a positive effect on milk flow physiology [26,43,61]. Although buffaloes with unstable milk let-downs are sometimes treated with exogenous oxytocin [25,43], we did not administrate exogenous oxytocin for pre-stimulation [25,43,61] of the OSF and NSF cows at milking. Accordingly, an increase in the MYM may be related to the decrease in residual milk, as it lets down the milk ejection easily [43]. Based on these reports, our results indicate that the SFAM could be a beneficial pre-stimulation for opening the teat canal and achieving complete milking [25] due to probably its impact on milking-related oxytocin secretion [26,43,61].

## 5. Conclusions

The SFAM enhanced the milk yield, fat content and FPR and reduced milk minerals and EC in indoor buffalo cows, regardless of parity. In addition, the PCA results indicate that four parameters (milk density value and lactose, SNF and protein contents) could be identified in the PC2 versus PC1 plot, explaining the relationship among the milk quality traits. It was concluded that the positive effects of SFAM on milk yield and some milk quality traits reflect the improvement in the cow’s welfare traits, such as TS, UHS and BCS. In the present study, the SFAM proved to be a beneficial milking strategy to enhance the welfare, health, behaviour, productivity, food quality and safety of lactating water buffaloes. Accordingly, our study could change consumer perceptions of buffaloes’ housing conditions and management in countries where buffalo breeding is critical in the economic and social field. In addition, due to their high quality, the OSF cows’ milk and dairy products (cheese, butter and cream) could be more appreciated by consumers and, as a result, actively promote the sustainable production of buffaloes. Further research should be focused on the pre-stimulation and milking-related oxytocin secretion effect of the SFAM for opening the teat canal and achieving complete milking, as well as the parameters and profitability studied in buffalo cows.

## Figures and Tables

**Table 1:** Ingredient composition and proximate composition of the experimental total mixed ration.

Ingredient	% of Dry Matter	Proximate Composition	
Meadow hay	32	Crude protein (%)	15
Maise silage	20	Neutral detergent fiber (%)	35
Grass silage	8	Acid detergent fiber (%)	22
Concentrates	40	Metabolisable energy (Mcal kg^−1^)	2.75

**Table 2:** Meanings and the subjective scores of the welfare assessment traits, including temperament, udder hygiene and body condition.

Welfare Assessment Traits			Score
Temperament ^1^	Udder Hygiene ^2^	Body Condition ^3^	
Very slow-very calm (docile)	Entirely clean	Emaciated	1
Slow-calm (slightly restless)	Clean	Thin	2
Normal (restless)	Dirty	Average	3
Sensitive-aggressive (nervous)	Very dirty	Fat	4
Very sensitive-very aggressive (aggressive)	Manure encrusted	Obese	5

^1^ Adapted from the scoring systems for dairy cattle [30,31]. ^2^ Adapted from the scoring systems for dairy cattle [16,32]. ^3^ Adapted from the scoring systems for Murrah buffaloes [33] and dairy cattle [34].

**Table 3:** Means of welfare assessment traits of buffalo cows according to supplementary feeding at milking and parity.

Main Factors		Welfare Assessment Traits		
SFAM	Parity	TS	UHS	BCS
OSF	2nd	1.21	1.42	2.86
	≥3rd	1.26	1.31	3.09
NSF	2nd	2.68	1.89	2.56
	≥3rd	2.47	2.21	2.67
SFAM				
	OSF	1.23	1.36	2.98
	NSF	2.57	2.05	2.61
Parity				
	2nd	1.94	1.65	2.71
	≥3rd	1.86	1.76	2.88
	SEM	0.137	0.089	0.048
*p*-value				
	SFAM	<0.001	<0.001	<0.001
	Parity	0.735	0.517	0.064
	SFAM × Parity	0.573	0.197	0.500

SFAM—supplementary feeding at milking; OSF—offered supplemental feeding at milking; NSF—not offered supplemental feeding at milking; SEM—standard error of the mean; SFAM × Parity—interaction between supplementary feeding at milking and parity; TS—temperament score, scale from 1 = very slow-very calm (docile) to 5 = very sensitive-very aggressive (aggressive); UHS—udder hygiene score, scale from 1 = entirely clean to 5 = manure encrusted; BCS—body condition score, scale from 1 = emaciated to 5 = obese.

**Table 4:** Means of milk yield per milking, milk components and physical traits of buffalo cows according to parity supplementary feeding at milking.

Main Factors		MYM (kg)	Milk Components						Milk Physical Traits		
SFAM	Parity		Fat (%)	SNF (%)	Protein (%)	FPR	Lactose (%)	Mineral (%)	Density (mg/mL)	FP (°C)	EC (mS/cm)
OSF	2nd	3.52	9.48	10.51	4.66	2.03	5.01	0.60	1.02	−0.72	3.57
	≥3rd	3.72	10.16	10.50	4.71	2.21	5.03	0.60	1.02	−0.72	3.53
NSF	2nd	2.60	7.69	10.94	4.82	1.60	5.11	0.66	1.03	−0.76	4.42
	≥3rd	2.55	7.50	10.33	4.54	1.64	4.91	0.66	1.03	−0.70	4.78
SFAM											
	OSF	3.62	9.82	10.51	4.68	2.12	5.02	0.60	1.03	−0.72	3.55
	NSF	2.57	7.59	10.63	4.68	1.62	5.01	0.66	1.03	−0.73	4.60
Parity											
	2nd	3.06	8.58	10.72	4.74	1.82	5.06	0.63	1.03	−0.74	3.99
	≥3rd	3.13	8.83	10.42	4.62	1.93	4.97	0.63	1.03	−0.71	4.16
	SEM	0.091	0.207	0.159	0.074	0.044	0.069	0.009	0.001	0.009	0.109
*p*-value											
SFAM		<0.001	<0.001	0.702	0.993	<0.001	0.931	0.001	0.108	0.646	<0.001
Parity		0.605	0.449	0.340	0.442	0.117	0.506	0.945	0.131	0.187	0.378
SFAM × Parity		0.368	0.188	0.355	0.293	0.345	0.429	0.989	0.546	0.109	0.268

SFAM—supplementary feeding at milking; OSF—offered supplemental feeding at milking; NSF—not offered supplemental feeding at milking; MYM—milk yield per milking; SNF—solids-not-fat; FPR—fat-to-protein ratio; FP—freezing point; EC—electrical conductivity; SEM—standard error of the mean; SFAM × Parity—interaction between supplementary feeding at milking and parity.

## Data Availability

The data supporting results reported here are available at a reasonable request from the corresponding author. The data are not publicly available because of privacy and ethical concerns.
